# Decision theory applied to image quality control in radiology

**DOI:** 10.1186/1472-6947-8-51

**Published:** 2008-11-13

**Authors:** Patrícia S Lessa, Cristofer A Caous, Paula R Arantes, Edson Amaro, Fernando M Campello de Souza

**Affiliations:** 1 Instituto Israelita de Ensino e Pesquisa Albert Einstein, São Paulo, SP, Brasil; 2 Neuroimagem Funcional, LIM 44, Universidade de São Paulo, FMUSP, São Paulo, SP, Brasil; 3 Eletrônica e Sistemas, Universidade Federal de Pernambuco, Recife, PE, Brasil

## Abstract

**Background:**

The present work aims at the application of the decision theory to radiological image quality control (QC) in diagnostic routine. The main problem addressed in the framework of decision theory is to accept or reject a film lot of a radiology service. The probability of each decision of a determined set of variables was obtained from the selected films.

**Methods:**

Based on a radiology service routine a decision probability function was determined for each considered group of combination characteristics. These characteristics were related to the film quality control. These parameters were also framed in a set of 8 possibilities, resulting in 256 possible decision rules. In order to determine a general utility application function to access the decision risk, we have used a simple unique parameter called *r*. The payoffs chosen were: diagnostic's result (correct/incorrect), cost (high/low), and patient satisfaction (yes/no) resulting in eight possible combinations.

**Results:**

Depending on the value of *r*, more or less risk will occur related to the decision-making. The utility function was evaluated in order to determine the probability of a decision. The decision was made with patients or administrators' opinions from a radiology service center.

**Conclusion:**

The model is a formal quantitative approach to make a decision related to the medical imaging quality, providing an instrument to discriminate what is really necessary to accept or reject a film or a film lot. The method presented herein can help to access the risk level of an incorrect radiological diagnosis decision.

## Background

The X-Ray is the main investigation method for the human body in various clinical conditions. Therefore, quality control procedures were implemented to standardize and define the minimum requirements for a gold standard radiology service. These requirements are worldwide reviewed by health institutions, including Brazilian National Institute of Health (Secretaria de Estado da Saúde, Centro de Vigilância Sanitária and Ministério da Saúde), as well as international institutions (International Commission on Radiological Protection – ICRP, World Health Organization – WHO, and International Commission of the European Communities – ICRU) monitoring the radiology practice [1].

The main idea behind the regulations in X-Ray application is to protect patients from radiation injury. The basic principles can be summarized as those described by ICRP 73 [2]: ionization radiation should be applied in cases where the benefits overcome the potential hazards. The objective is to maximize the gains over the potential harms using procedures to protect against radiation. The applications of these two principles are the main goal of a Quality Control (QC) program: apply the minimum required radiation to maintain the diagnostic value of the images. Radiation must be controlled and the radiological images should have always enough quality to provide the required information.

Excellence in imaging quality standards is achieved through published guidelines of radiological societies (e.g. Radiological Society of North America guidelines). There are standard procedures for each exam that the technologist knows and follows when acquiring an image. There are also general rules related to examination steps and acquisition device settings. Every step is dependent of the patient's characteristics, such as height. In addition, some issues are difficult to standardize, although the major of technical procedures were monitored for a proper quality assurance [3]. These non-measurable factors usually have a negative impact in the standardization of radiological practice with undesirable effects. A preliminary study made between 1992 and 2003 in Brazil showed that 5.15% of the films were rejected films; resulting in patient's re-expositions, equipment lifetime reduction and an overall increased operational cost. The main cause of film loss identified in this study was lack of standardization [4].

A QC program is typically a result of multidisciplinary interaction, involving several steps: habitual verification of technical parameters in X-ray equipments, systems registry (films, digital cassettes) and other procedures used to deliver the images for medical interpretation. The definition of QC also includes a series of standardized tests developed to detect changes in X-ray equipments [5,6]. The final result of a successful QC should primarily be a reduction in the number of re-exposed or over-exposed patients and a high quality imaging diagnostic. Also, it should provide minimal financial costs and increase the equipment lifetime. This usually requires a complex rule of decisions, usually made by the physicists, engineers and radiologists in a joint effort to maximize two principles: improve the image quality and reduce radiation exposure.

Decision Making Theory (DMT) offers an integrated framework to mathematically define a preference and/or probability model for each step of the QC program. Priority of preference in sets of possible outcomes and states of information (uncertainty or imprecision) are represented as distribution possibilities [7] and therefore statistically analyzed. This formulation allows a more direct measurement of the outcomes from a certain decision and has been implemented successfully in many different contexts. One of the most used implementations of DMT was proposed by Von Neumann and Morgenstern [8]. These authors modeled the quantitative utility function based on a set of axioms that described decision-maker's behavior. Various implementations made in medicine were based on their approach with practical examples [9,10]. Their work has demonstrated the importance of this kind of framework according to a decision policy regarding a case of a preoperative patient management care before opting for a major elective surgery. All available information to decision-making, through informed priors, allow the appropriate decisions to be made [10].

In essence, what the decision theory formalizes is the common sense idea that an individual should take the best action based upon what he or she wants, knows, and can do [11,12]. In a radiology routine, it is the technician who acquires the image and observes it initially. It is often up to them – without ever asking the radiologist – whether or not another image must be taken due to poor quality acquisitions. It should also be noted that the technologists often acquire an image with certain parameters because they know which radiologists will evaluate the exam and what their personal preferences are. The classification of this "individual" choice depends on direct or indirect aspects. The person who makes this decision could be the administrator of the radiology service department, the radiologist itself, the technician or even the patient when refusing to perform a second or third exam in order to reacquire the image.

The basic parameters of decision theory involve some sets, probabilistic mechanisms, and decision rules [13].

The four main sets are:

1) a payoff set (p) – the offered benefit in a specific situation, consisting of all the possible consequences of the actions to be taken;

2) a set of "states of nature" (θ) – containing all the possible situations of the study; typically unknown parameters in the phenomena (i.e. if a film has a good or bad quality) involved;

3) a set of observations (x) – relating to the states of nature (i.e. spatial resolution, sensitivity of an image);

4) a set of possible courses of action (a) – to reject or accept the film.

The three main probabilistic mechanisms are:

*1) The consequence function – P*(*p*_*i*_|*θ*_*j*_, *a*_*k*_). It shows the distribution probability of the payoffs for each pair of defined events – "state of nature" vs. action. It is necessary to consider the utility function, which represents the preferences of the decision maker in situations of uncertainty. In order to reach the model of utility function (u), a process of eduction is applied to prioritize the good and bad actions strategies incorporated into a unique evaluation dimension. This function is a polynomial one with a degree called *r*. The higher the *r *value, the most complex will be the utility function.

*2) The distribution probability (likelihood function) – P*(*x*_*i*_|*θ*_*j*_). According to the determined observations of the state of nature, a film can have a good or bad quality including its own characteristics (contrast, speed, fog, digital exposition, latitude and other). The distribution probability considers the correlation between the observations made and the state of nature.

*3) A prior distribution – π*(*θ*_*j*_). Possibly elicited from an expert, regarding to the state of nature. If the institution maintains a database of QC, it is possible to estimate a prior distribution to be applied to the test.

The decision rule (*d*) associated for each observation (possible actions to be taken):

1) The deterministic choice is the most simple decision rule for each observation in which an ideal action should be taken.

2) The randomized decision rule yields two other types: 2.1) The randomization upon the non-randomized rules before any observation is made.

2.2) The behavioral randomized rule *b*(*a*_*j*_|*x*_*i*_), in which a first observation is made. After this observation, a randomization of all possible actions is considered. The behavioral rule is more powerful, in the sense that it can access any risk; this is not the case regarding the randomized rule.

The risk function (*Rd*) compares the decision rules and the consequence function is defined by the process for obtaining the probability of a certain result related to the "state of nature" (in our case, a good or bad image). Based on the consequence function the decision-maker chooses, a certain proceeding or action is taken. This probabilistic mechanism should be better understood when analyzed together with the decision theory techniques. It can be inferred from an available database, from the elicitations of an expert or from both.

Herein, we suggest a general guideline, based on the decision-making paradigm, as proposed by the report from ICRU in 1996 [1]. According to this report, the techniques of statistical DMT should be used as a standard procedure to treat problems of image detection, edge detection, noise and sharpness. The parameters referred by the present work are: inadequate use, inadequate sensibility and/or inadequate spatial resolution related to the radiological chain process. The application of the decision making theory was restricted to acquired images, not considering other parameters that could interfere before the image detection. The final outcome considered was to accept of reject a film lot. With this new approach, there are good consequences like: the patient's satisfaction, a correct diagnosis and stable financial costs.

This work does not aim on a new method proposition to improve the sensibility or specificity of the data acquisition system. The approach also applied a very well known concept of the radiology practice: the Receiver Operating Characteristics, also known as the ROC curve. This curve is determined according to a set of probabilities: true positive = P (positive test/positive for disease) vs. false positive = P (positive test/negative for disease). In other words, sensibility vs. a false positive or P (altered exam/has the disease) vs. P (altered exam/has not the disease) parameters that are part of most papers published in radiology journals, in which the accuracy of a diagnostic imaging test is verified.

This is the TPF (function of true positive probability) and FPF (function of false positive probability) ROC curve. It has a purely inferential character, and is a very particular case of a more general ROC curve, where the loss function is the ideal observer loss function (i.e., the negative of the utility function). We have four possibilities: if the film is bad, we reject it; if the film is good, we accept it; if the film is bad and we accept it, and finally, if the film is good and we reject it. In the first two cases, the target was reached. In the latest the target was missed, and they correspond to errors. In mathematical terms, the loss function is given by:

*L*(*θ*_*i*_, *a*_*j*_) = -*u*(*θ*_*i*_, *a*_*j*_)

The ideal observer loss function is given by:

*L*(*θ*_*i*_, *a*_*j*_) = 0 if *a*_*i *_= *θ*_*j*_; *L*(*θ*_*j*_, *a*_*i*_) = 1 if *a*_*j *_≠ *θ*_*i*_, *i*, *j *= 0, 1.

This means that if we reach the target, we have no fine (the loss is null). If we miss the target, we pay a fine equal to one (the loss is equal to 1). But this rather peculiar utility function does not reflect the preferences of a decision-maker, since the negative of the ideal observer loss function is just an inference criterion.

When the ideal loss function is applied, the risks were calculated by:

*Rd*(*θ*_*j*_) = -Σ*P*(*x*_*i*_|*θ*_*j*_)*u*(*θ*_*j*_, *d*(*x*_*i*_))

These probabilities correspond to the specificity, sensitivity, false positive and false negative conditions. In these inferential contexts, the classical TPF and FPF ROC curve is the typically presented as the true positive versus the false positive conditions, with a 0–100% scale on both axes (concave function). In the current work, since we are dealing with preferences, the risks will not be considered as probabilities. The axes of the ROC curves will have a scale corresponding to the applied utility function, which should be educed from the decision-maker and represents his preferences (convex curve). It is the locus of all possible Neyman-Pearson Rules and not a TPF- FPF ROC curve.

The ROC curve axis reflects the preference dimension of the decision-maker regarding to the data acquisition system (i.e. technology or radiography type) and will imply in a likelihood function. This construction is independent from a considered database and the probability will usually be very similar.

In order to accept or reject a film lot according to this procedure is necessary to obtain the correct diagnosis, the cost optimization and the client's satisfaction. The client's satisfaction will be measured by the utility function, which is different from the one that corresponds to the ideal observer loss function. All values of probable observations were proposed based on previous information from real datasets and hypothetical predictions applications. The investigation researched some possibilities according to the dependency values related to the image quality assurance. In summary, this is a case in which the decision theory is used to establish a framework for radiographic film quality control protocol; this is done by studying the consequence of an action as a function of a specific probability distribution over possible consequences.

## Methods

The decision making theory was implemented for specific computational tools. The data was stored in a Quattro Pro v. 9.0 (Borland Inc – USA) and in a Statistica v. 5.3 (StatSoft – USA). All data was obtained from densitometry data of the radiographic films of a phantom obtained by a digital densitometer (Model 07–443, Nuclear Associates Victoreen 07–443 Clamshell, United States). The values of the film characteristics were calculated from 15 different measures of radiation intensity.

The images were made in a conventional X-Ray, Medicor 500 mA and 125 kV, from the Hospital das Clínicas da Universidade Federal de Pernambuco (HC-UFPE), Brazil. The values were obtained from data analysis of the radiographic film (characteristic curve, modulated transfer function and the Wiener spectrum as exposed in ICRU [1]). Attributes such as contrast, latitude, speed and fog were determined according to our observations from each film condition (bad quality or good quality).

In case of two possible actions (accept/reject) and no prior probability, we applied the behavioral randomized [7] and Neyman-Pearson rules. In case of two possible actions, the observation sample space was partitioned into two regions, one of rejection and the other of acceptance. The model is very simple in compliance with Ockham's razor principle. The consequence function was calculated based on the subjective preference of the person making the decision.

## Results

**Figure 1:**
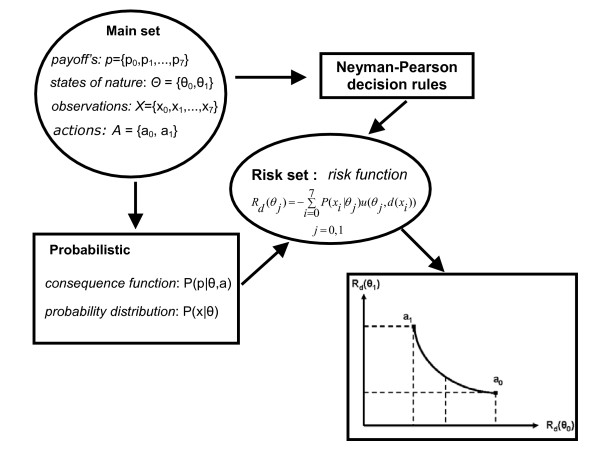
Schematic representation of the Decision Making Theory applied to the radiographic film quality control parameter.

The first approach will consider a dichotomy in each of the three attributes: diagnosis, cost and client satisfaction, where eight possible payoffs cases (see figure 1) will be considered:

*p*_0 _= (*incorrect diagnostic, high cost, low client satisfaction*)

*p*_1 _= (*incorrect diagnostic, low cost, low client satisfaction*)

*p*_2 _= (*incorrect diagnostic, high cost, high client satisfaction*)

*p*_3 _= (*incorrect diagnostic, low cost, high client satisfaction*)

*p*_4 _= (*correct diagnostic, high cost, low client satisfaction*)

*p*_5 _= (*correct diagnostic, low cost, low client satisfaction*)

*p*_6 _= (*correct diagnostic, high cost, high client satisfaction*)

*p*_7 _= (*correct diagnostic, low cost, high client satisfaction*)

Based on the parameters described above, a set of "states of nature" was established. A film lot is considered good if the percentage of bad units from a film box is below a certain pre-specified value. There is a natural doubts of which of these states reflect a true condition scenario.

This uncertainty establishes the decision problem. Where *θ *represents the real "state of nature" of the film, we have:

*θ*_0 _= the film is a bad quality one;

*θ*_1 _= the film is a good quality one.

The space or set of observations should consider a finite number of possibilities. We considered three groups of attributes: film usage, film sensibility and spatial resolution.

For each situation of film usage, film sensibility and spatial resolution, a dichotomy was considered, based on the limits of the values dependency. Another set (eight possibilities) was originated for the following observations:

*x*_0 _= (*inadequate for use, inadequate sensibility, inadequate spatial resolution*)

*x*_1 _= (*inadequate for use, inadequate sensibility, adequate spatial resolution*)

*x*_2 _= (*inadequate for use, adequate sensibility, inadequate spatial resolution*)

*x*_3 _= (*inadequate for use, adequate sensibility, adequate spatial resolution*)

*x*_4 _= (*adequate for use, inadequate sensibility, inadequate spatial resolution*)

*x*_5 _= (*adequate for use, inadequate sensibility, adequate spatial resolution*)

*x*_6 _= (*adequate for use, adequate sensibility, inadequate spatial resolution*)

*x*_7 _= (*adequate for use, adequate sensibility, adequate spatial resolution*)

The problem considered in this case had only two possibilities of action:

*a*_0 _= to reject the film lot;

*a*_1 _= to accept the film lot.

The effective action related to the condition of the state of nature reflects the probability of the feasible outcomes.

This probabilistic mechanism depends on the technology of the data acquisition system (sensors, digitizers and others). A plausible set of probabilities for this problem was shown in table 1. These values correspond to an estimated distribution of the combined information mentioned above obtained from radiologists and physicists involved in the QC program.

**Table 1:** The film usage, film sensibility and spatial resolution attributes provide the likelihood function (*P*(*x*_*i*_|*θ*_*j*_)) of the analyzed images related to the values dependency.

**P(x_i_|θ_j_)**	**x_0_**	**x_1_**	**x_2_**	**x_3_**	**x_4_**	**x_5_**	**x_6_**	**x_7_**
***θ***_*0*_	0.41177	0.16807	0.14706	0.11345	0.07563	0.05042	0.02521	0.0084
***θ***_*1*_	0.00977	0.01303	0.03257	0.06515	0.09772	0.13029	0.16287	0.4886

These values correspond to an estimated distribution of the combined information obtained from radiologists and physicists involved in the quality control program.

The likelihood function *P*(*x*_*i*_|*θ*_*j*_) consists of two values, in which *i *corresponds to the observed value and *j *to the true value. The value of *x*_*i *_(a set of observations) represents what is obtained from the equipment, and *θ*_*j *_(a set of "states of nature") is the "true" value: a good or bad film quality.

The consequence function is the probability of obtaining certain payoff regarding the "state of nature" and a specific action of the decision-maker. Table 2 shows the probabilities for each pair (*θ*_*i*_, *a*_*j*_) of a consequence function. These values where calculated with data from Table 1. For instance, regarding *x*_*o*_*("inadequate for use, inadequate sensibility, inadequate spatial resolution") *the probability of each consequence (*p*_*0 *_- *p*_*7*_) if the action is to reject (a_*0*_) the film can be obtained from Table 2. If the film is bad (*θ*_*o*_), the highest probability is *p*_4 _(0.300), which means that there is a high chance of making the right diagnosis. A high cost is involved in this case due to the necessity of another film, trial procedure and professional staff time. As a consequence, the client satisfaction in the process is compromised since the patient will probably repeat the procedure, and be re-exposed to the X-rays.

**Table 2:** The probabilities are shown for each pair (*θ*_*i*_, *a*_*j*_) of a consequence function.

**P(x_i_|θ_j_, a_k_)**	**p**_**0**_	**p**_**1**_	**p**_**2**_	**p**_**3**_	**p**_**4**_	**p**_**5**_	**p**_**6**_	**p**_**7**_
***(θ*_0_, *a*_0_*)***	0.150	0.110	0.070	0.050	0.300	0.200	0.080	0.040
***(θ*_0_, *a*_1_*)***	0.300	0.250	0.150	0.100	0.060	0.040	0.080	0.020
***(θ*_1_, *a*_0_*)***	0.100	0.060	0.100	0.150	0.100	0.050	0.250	0.190
***(θ*_1_, *a*_1_*)***	0.050	0.060	0.090	0.060	0.110	0.130	0.200	0.300

All values where calculated with the data obtained from table 1. The highest probability of a correct diagnosis with an increased cost and low client satisfaction was represented by *P*(*p*_4_|*θ*_0_,*a*_0_) = 0.30.

### The decision rules

The "state of nature" and the problem solution were obtained by the rule of Neyman-Pearson. The decision-maker utility function enunciated the level *θ *of tolerated risk when *θ*_0 _was considered the true "state of nature".

We have applied two *r *values to simulate the decision-maker's influence or preference upon the decision rule to adopt an optimized action (ideal). The *r *value is actually the polynomial grade used in the mathematical formulation. We chose *r *= 2 and *r *= 4, in order to have the classical quadratic loss function (from Theory of Decision Making) and a more complex formulation. This was done in order to check whether the results would be the same, regardless of the decision-making opinion.

The observations were considered from a set of 8 possibilities as shown in Table 1. Thus, we have 256 possible decision rules (2 actions with 8 established observations). In order to determine a general utility function to access the decision risk, we have used just one parameter – *r *– in which the utility function considered was *u*(*p*) = *p*^*r*^.

Two values of *r *were used: *r *= 2 and *r *= 4. The higher values of *r *reflect an increased risk of the decision-maker. If the patient had to decide about the rejection or acceptance, he or she would tend to be more adverse to risk. We chose *r *= 2 as a conservative risk behavior simulation to the patient choice, so that, the values of *P*(*x*_*i*_|*θ*_*j*_) = probability values of the signal/noise ratio were relayed on imaging equipments and its components (see Table 1) and *P*(*p*_*i*_|*θ*_*j*_, *a*_*k*_) = once the nature condition of the chosen data *θ*_0_(*bad film*) or *θ*_1_(*good film*) regarding to *a*_0 _= *to reject *or *a*_1 _= *to accept *action (data not shown). The consequence function is defined as *p*_*i *_(payoffs), under probability of *P*(*p*_*i*_|*θ*_*j*_, *a*_*k*_) (see Table 2).

**Figure 2:**
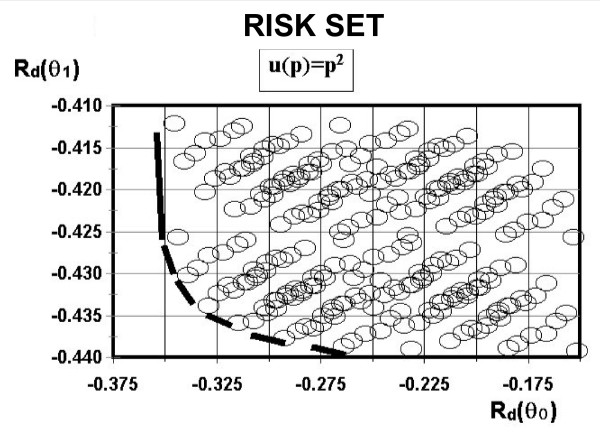
**The risk set for the case *r = 2*.***R*_*d*_(*θ*_0_) – Risk set for a bad film quality and *R*_*d*_(*θ*_1_) -Risk set for a good film quality. *u*(*p*) = *p*^2 ^– Utility function to use just one parameter (*r *= 2).

**Figure 3:**
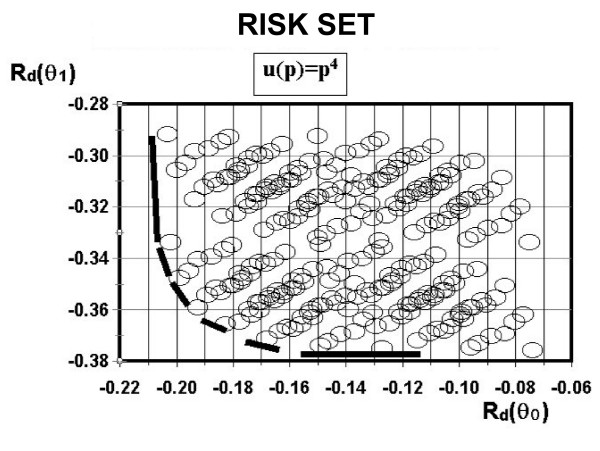
**The risk set when *r = 4*.***R*_*d*_(*θ*_0_) – Risk set for a bad film quality and *R*_*d*_(*θ*_1_) – Risk set for a good film quality. *u*(*p*) = *p*^4 ^– Utility function to use just one parameter (*r *= 4).

The values shown in figures 2 and 3 are a simple plot of the ROC obtained from all possible Neyman-Pearson decision rules results. The observation of those figures depicts the low risk level of the d_16 _decision rule. In other words, if the decisor-maker chooses d_16_, a low probability situation (related to the risk) of rejecting a good film or accepting a bad one would be the plausible decision. The decision rule d_16 _suggests that the decision-maker will refuse all observations including the "inadequate for use"(*x*_0 _- *x*_3_) parameter.

### Simulations with the model

The main purpose of the simulation is to analyze the influence of the utility function on the decision rule to accept or reject the film lot. There are several possibilities concerning the emphasis and accuracy on various aspects of the possible outcomes, contemplating the quality control protocol. In particular, the characteristic of risk aversion was evaluated. The probability of *x*_0 _= (inadequate for use, inadequate sensibility, inadequate spatial resolution) when *θ *("states of nature"), *θ*_0 _(*bad film*) is 0.41177 (worst scenario) – case in which the report is incorrect and the patient re-exposure will be necessary – the probability of *x*_0 _is 0.00977, if *θ *("states of nature") is *θ*_1 _(*good film*). On the other hand, the probability of when *θ *("states of nature"), *θ*_0 _(*bad film*) is 0.0084 makes the probability of *x*_7 _be 0.4888 of *θ*_1 _(*good film*).

Table 2 demonstrates all considered consequence functions. The highest probability of a correct diagnosis with an increased cost and low client satisfaction was represented by *P*(*p*_4 _| *θ*_0_, *a*_0_) = 0.30 (Table 2).

Consider the utility function $u(\theta_{0},a_{0\;or\;1})=\sum_{k=0}^{7}u(p_{k})P(p_{k}|\theta_{0},a_{0\;or\;1})$ and $u(\theta_{1},a_{0\;or\;1})=\sum_{k=0}^{7}u(p_{k})P(p_{k}|\theta_{1},a_{0\;or\;1})$. In order to calculate the risk function *R*_*d*_(*θ *_0_) and *R*_*d*_(*θ*_1_) corresponding to each utility function (polynomial degree) *r*, we used $R_{d}(\theta_{0})=-\sum_{i=0}^{7}P(x_{i}|\theta_{0})u(\theta_{0},a_{0\;or\;1})$ and $R_{d}(\theta_{1})=-\sum_{i=0}^{7}P(x_{i}|\theta_{1})u(\theta_{1},a_{0\;or\;1})$, presented as *R*_*d*_(*θ*_0_) × *R*_*d*_(*θ*_1_) in figure 2, with *r *= 2 and in figure 3 with *r *= 4.

Tables 1 and 2 correspond to the likelihood and consequence functions, respectively. In addition, Table 3 represents the results obtained with the Neyman-Pearson decision rules for two values of *r*. Simulated values were used to calculate the risks from decision rules for each observation and also to determine a ROC analysis for each risk set.

**Table 3:** Representation of the results accomplished with the Neyman-Pearson decision rules for two values of *r*.

**r**	**Regra**	**x**_**0**_	**x**_**1**_	**x**_****2****_	**x**_**3**_	**x**_**4**_	**x**_**5**_	**x**_**6**_	**x**_**7**_	**R**_**d**_**(θ**_**0**_**)**	**R**_**d**_**(θ**_**1**_**)**
2	d_16_	a_0_	a_0_	a_0_	a_0_	a_1_	a_1_	a_1_	a_1_	-0.31	-0.44
2	d_192_	a_1_	a_0_	a_1_	a_1_	a_1_	a_1_	a_1_	a_1_	-0.18	-0.44
4	d_16_	a_0_	a_0_	a_0_	a_0_	a_1_	a_1_	a_1_	a_1_	-0.18	-0.36
4	d_192_	a_1_	a_0_	a_1_	a_1_	a_1_	a_1_	a_1_	a_1_	-0.10	-0.37

Results of the simulation for a parameter (*r*) to a determined decision rule (*d*); *R*_*d*_(*θ*_0_) – Risk set for a bad film quality; *R*_*d*_(*θ*_1_) – Risk set for a good film quality.

In figure 3, the decision rule *d*_16 _corresponds to the vertex of the curve when the risk *R*_*d*_(*θ*_0_) is set to *r *= 4. This rule says that if the observation was inadequate for use, the film must be rejected. All other options were accepted (see Table 3). This rule corresponds to *R*_*d*_(*θ*_0_) = -0.18 and *R*_*d*_(*θ*_1_) = -0.36. When *r *= 2 for the same value, we have *R*_*d*_(*θ*_0_) = -0.18 and the minimum value in the curve corresponds to *d*_192 _(*R*_*d*_(*θ*_1_) = -0.44). This rule expresses that the only rejection condition for the film is when usage and sensibility are inadequate and spatial resolution is adequate (Figure 2).

However, when *r *= 4 (*d*_192_) the risk of accepting a bad film is increased (*R*_*d*_(*θ*_0_) = -0.18) and the risk of accepting a good film increases as well (compared to *d*_16_). This rule was not the ideal one since it rejects the finest option: adequate for use, sensibility and spatial resolution.

## Discussion

The problem of accepting or rejecting a radiographic image based on the decision theory method was modeled here. The decision theory was applied on a hypothetical model. The ROC curve axis represent risks levels based on a preference concept or choice determined by the eduction of the utility function von Neumann-Morgenstern – this curve represents how a set of decision rules behave related to the risk of a consequence set. The ROC analysis for each risk set is a graphic representation, common to radiologists, and provides a manner to decide how a decision rule could be used, according to the false positive or sensitivity rate (Neyman-Pearson decision rule [14]).

In modern radiological services, it is extremely difficult to manage quality control processes. The mathematical approach can help, dealing with complex decisions such as reject or accept a radiographic image. The main result obtained in this modeling exercise was provided by a decision rules set that minimizes the risk of an undesired consequence. In addition, the solution proposed at this point can be applied in a practical radiology routine.

Residents and non-professionals staff of the radiology field have to learn how to make decisions, generally from gathering information from other more experienced professionals and from literature information. Decision Theory offers a way to arrange decisions in a rational manner, thus, avoiding many risks that may affect the patient health quality. If one radiologist considers an image appropriate for radiological appraisal, many possibilities may be influenced by this decision. Besides, the level of consequences depends on the type of the clinical question. If the radiography is performed to diagnose a broken arm, the presence of an insignificant spot in the image is generally not relevant as in the case of a slight spot in mammography exam performed for tumoral screening. The presence of micro-calcifications with specific morphological aspects has a direct impact on the next step, which may include or not additional invasive procedures. Therefore, the probability of a correct diagnosis could be directly affected by the quality of a simple X-ray image.

The expense of an image production is increased if a film does not have a good quality. X-ray re-exposure will be inevitable and the patient will certainly be unsatisfied at this point. This behavior variable should not be misguided with a simple level of comfort. The client contentment must be contemplated, not only by the correct diagnosis, but also by the number of necessary radiographic trials in the service. As a presumable consequence, the service could lose a representative number of clients, which would represent a substantial loss in profits.

All radiology services have an administrative design for quality control (QC) of the radiographic film. This is emphasized by the ICRU and is generally based on gold standard guidelines and limits of technical parameters among the used values to access the film quality. Although there are similar procedures reported in the literature [15], none of them is based on a formal quantitative model. Generally, the result of a QC program is measured by the number of exams correctly performed, costs involved and the patient's satisfaction. These variables are not easily measured beyond descriptive statistics. The decision is commonly not settle on a formal quantitative model, but in personal experience and historical background of the institution. The management team is responsible to decide upon the equipment choices variety, imaging procedures, questionnaires and personal training programs.

The use of Bayesian statistics allows one to give a closer look at the real based management information [14,16,17]. Historical information was considered and used for a prior distribution of the probabilities as a mean to avoid fake or unlikely occurrences [18,19]. This approach can be applied in distinct radiological scenarios. This concept or perception adapted to other settings of variables could include another image modality, clinical context and/or strategies for a perspective research of decisions or administrative managing.

The main challenge concerning the decision theory implement is to define the state of nature (good or bad quality of the radiographic film). Therefore, a given action can lead to a favorable payoff of the decision-maker whatever the state of nature. If the state of nature is a good one, the preferred result would be more likely to occur. Even so, a determined action for a situation could probably unfold as a complete disaster in the case of a very unfavorable payoff, while the true state of nature is completely different or wrong. Note that in two other cases, still with a less significant extent, positive probabilities have appeared. The consequence of an action must be established through a numeric scale according to the decision-maker preferences [13].

Possible consequences were summarized based on the quality of an image in three distinct aspects: diagnosis (correct or incorrect), cost (high or low) and the patient satisfaction (high or low). In summary, the proposed decision problem is: having one of the eight possibilities (*x*) what decision should be made regarding the value of (*θ*) so that it can be considered true? The person responsible for the radiology department or the radiology management team (usually the decision-maker) will adopt a criterion for the decision process in order to guide the choice that will minimize the risk of having an undesired consequence. The choice of the vector *x *(a set of observations) and of the vector *θ *(a set of "states of nature") will depend on the considered precision degree as well as on the reliable technology. The consequence function *P*(*p *| *θ*, *a*) was estimated from the data obtained from the experiments or from a historical service database. It includes more probabilistic mechanisms than those found in a film. The utility function's eduction was not determined by any decision-maker. It is important to mention that the proposal of probabilistic mechanism in the likelihood function can be adjusted to any information provided. As a consequence the data consistency will depend on the source confidence. A consequence (*p *– a set of payoff), as described in the results, was related to the set of payoffs involved in a right or wrong diagnosis, a higher or lower cost and higher or lower client satisfaction, independently of the technology or of the radiologist that made the diagnosis. Each pair (*θ*, *a*) has a specific risk correlation to the preferences of the decision-maker. The decision-maker preferences, *per se*, were dictated by a behavior parameter in a risky circumstance. Decision theory application estimates the risks for all possible rational choices under uncertainty and allows a rational decision for all computed information during a specific procedure (Bayesian theory).

Again, the choice of the best decision rule can be found at the vertex region of the curve in the risk set *R*_*d*_(*θ*_0_) with *r *= 4 which is a result of the 256 possible combinations, as described previously. The corresponding graphic (Figure 3) information corresponds to *d*_16_, which means that the observation was inadequate for use (bad film), and the film was rejected (Table 3). The decision-maker option was to accept it. When adopting the criterion mentioned above, we found that the minimum risk combination of *R*_*d*_(*θ*_0_) × *R*_*d*_(*θ*_1_) corresponds to (Figure 3) *R*_*d*_(*θ*_0_) = -0.18 when the state of nature of the film was bad (*θ*_0_), and the risk of the film was considered good (*θ*_1_) with *r *= 4 corresponding to *R*_*d*_(*θ*_1_) = 0.36. In the case of *r *= 2, if the decision-maker had chosen the same value *R*_*d*_(*θ*_0_) = -0.18, the decision rule would be different: the film is rejected when the state of nature and its technical quality are bad -*d*_192_. Thus, the minimum risk combination *R*_*d*_(*θ*_0_) × *R*_*d*_(*θ*_1_) is not the best decision rule for this case. This model can be refined by constructing *p, θ, x*, values which may assume any mathematical (finite or infinite, discrete or continuous, scale or vectorial) representations. There are no restrictions for the current adopted classification.

## Conclusion

The proposed model allows a cost-utility analysis of the radiographic film quality control. The decision theory addressed to this quality control issues established a systematic procedure, a well defined protocol approach and suggested a better use of the available imaging technologies acquisitions. Moreover, depending on the utility function, it allows us to analyze how the patient choices or the radiographic service decision-making preferences affect the decision rule. It constitutes a paradigm not only for a film quality control, but also for the radiographic service quality control in an integrated framework.

## Competing interests

The authors declare that they have no competing interests.

## Authors' contributions

PSL developed this work as a part of her Ph.D. thesis; she designed the study, analyzed the data and is responsible for the manuscript preparation. CAC and PA contributed to ideas, to the organization and to the writing of the manuscript with PSL. EAJ contributed to the discussion for application in radiological images and to the overall manuscript preparation. FMCS was PSL's supervisor and mentor during her PhD program, suggesting the subject of use of image analysis in radiology in the context of decision making theory.

## Pre-publication history

The pre-publication history for this paper can be accessed here:


